# The cardioprotective effects of secoisolariciresinol diglucoside (flaxseed lignan) against cafeteria diet-induced cardiac fibrosis and vascular injury in rats: an insight into apelin/AMPK/FOXO3a signaling pathways

**DOI:** 10.3389/fphar.2023.1199294

**Published:** 2023-07-11

**Authors:** Azza H. Abdelwahab, Amira M. Negm, Eman S. Mahmoud, Rania M. Salama, Mona F. Schaalan, Azza A. K. El-Sheikh, Basma K. Ramadan

**Affiliations:** ^1^ Physiology Department, Faculty of Medicine (Girls), Al-Azhar University, Cairo, Egypt; ^2^ Histology Department, Faculty of Medicine (Girls), Al-Azhar University, Cairo, Egypt; ^3^ Pharmacology and Toxicology Department, Faculty of Pharmacy, Misr International University, Cairo, Egypt; ^4^ Clinical Pharmacy Department, Clinical and Translational Research Unit, Faculty of Pharmacy, Misr International University, Cairo, Egypt; ^5^ Basic Health Sciences Department, College of Medicine, Princess Nourah bint Abdulrahman University, Riyadh, Saudi Arabia; ^6^ Medical Sciences Department, Faculty of Oral and Dental Medicine, Misr International University, Cairo, Egypt

**Keywords:** flaxseed, secoisolariciresinol diglucoside, apelin, dyslipidemia, vascular injury, cardiac fibrosis, cafeteria diet

## Abstract

**Introduction:** Fast food is a major risk factor for atherosclerosis, a leading cause of morbidity and mortality in the Western world. Apelin, the endogenous adipokine, can protect against cardiovascular disease via activating its receptor, APJ. Concurrently, secoisolariciresinol diglucoside (SDG), a flaxseed lignan extract (FLE), showed a therapeutic impact on atherosclerosis. The current study aimed to examine the effect of SDG on cafeteria diet (CAFD)-induced vascular injury and cardiac fibrosis via tracking the involvement of the apelin/APJ pathway.

**Methods:** Thirty male rats were allocated into control, FLE-, CAFD-, CAFD/FLE-, and CAFD/FLE/F13A-treated rats, where F13A is an APJ blocker. All treatments lasted for 12 weeks.

**Results and discussion:** The CAFD-induced cardiovascular injury was evidenced by histological distortions, dyslipidemia, elevated atherogenic indices, cardiac troponin I, collagen percentage, glycogen content, and apoptotic markers. CAFD increased both the gene and protein expression levels of cardiac APJ, apelin, and FOXO3a, in addition to increasing endothelin-1, VCAM1, and plasminogen activator inhibitor-1 serum levels and upregulating cardiac MMP-9 gene expression. Moreover, CAFD reduced serum paraoxonase 1 and nitric oxide levels, cardiac AMPK, and nuclear Nrf2 expression. FLE attenuated CAFD-induced cardiovascular injury. Such effect was reduced in rats receiving the APJ blocker, implicating the involvement of apelin/APJ in FLE protective mechanisms.

**Conclusion:** FLE supplementation abrogated CAFD-induced cardiac injury and endothelial dysfunction in an apelin/APJ-dependent manner.

## 1 Introduction

Atherosclerosis is the primary cause of ischemic heart disease. The etiology of atherosclerotic cardiovascular diseases is multifactorial and the most common risk factors include hypercholesterolemia, hypertension, diabetes mellitus, and cigarette smoking. Furthermore, other cardiovascular disease inducers include a sedentary lifestyle, central obesity, and high-caloric diets, especially that are high in saturated and trans-fatty acids (Shafi et al., 2019). The progression of atherosclerosis is enhanced by multiple pathological features including the excessive release of inflammatory mediators and reactive oxygen species (ROS), stimulation of growth factors, and the proliferation of vascular smooth muscle cells (Marchio et al., 2019). Moreover, overexpression of matrix metalloproteinases (MMPs) weakens atherosclerotic plaques, causing their rupture, and consequent myocardial infarction, and cerebrovascular diseases (Kattoor et al., 2017; Myasoedova et al., 2018). Emerging evidence suggests that dietary composition and overall diet quality improvement may prevent primary and secondary cardiovascular events. Hence, numerous strategies have been developed to reduce the incidence of cardiac injury to those who already have cardiac diseases and are at risk of recurrence (Yu et al., 2018).

The flaxseed (*Linum usitatissimum* L.) is an annual herb that belongs to the *Linaceae* family. Among the compounds that present biological activity, the phenolic compounds, lignans, are of special interest. Flaxseed is particularly the richest known source of lignans, with lignin production at 75–800 times that of other oil seeds. The principal dietary lignan present in flaxseed is secoisolariciresinol diglucoside (SDG) (Sangiorgio et al., 2023). Currently, there are reports about the beneficial effects of flaxseed or SDG on cardiovascular health, diabetes, and fatty liver highlighting the involvement of the antioxidant and anti-inflammatory effects in mediating its effects (Zanwar et al., 2014; Al Za’abi et al., 2021). As previously reported, SDG and its metabolites managed to reduce serum total cholesterol (TC), low-density lipoprotein-cholesterol (LDL-C), and other atherogenic indices (Prasad et al., 2020; Guo et al., 2021). Additionally, SDG cardiovascular protective effects can be attributed to the increased expression of vascular endothelial growth factor, and endothelial nitric oxide (NO) synthase, which enhanced myocardial angiogenesis (Zanwar et al., 2014). Both apelin, the endogenous adipokine, and its G protein-coupled apelin receptor (APJ) are involved in the regulation of cardiovascular functions, fluid homeostasis, vessel formation, cell proliferation, and food intake (Castan-Laurell et al., 2021). Moreover, apelin exerted a cardioprotective role against myocardial infarction, where the stimulated apelin expression increased cardiac output, lowered blood pressure, and attenuated hypertrophy (Chen et al., 2022).

The exact apelin-induced signaling pathways that are involved in cardiovascular pathology and the role of nutraceuticals and pharmacological agents on this pathway are still to be investigated. Synchronously, the cardioprotective impact of flaxseed or SDG in an experimental model that mimics high-calorie fast food-induced cardiac and vascular injury is still lacking. Thus, the present study was designed to investigate a novel cardioprotective impact of SDG, the flaxseed lignan extract (FLE), against cafeteria diet (CAFD)-induced cardiac and vascular injury in rats, highlighting the changes in the mechanistic redox-inflammatory-apoptotic tracks and the involvement of the apelin/APJ signaling pathway.

## 2 Materials and methods

### 2.1 Ethics statement

All experimental procedures and animal husbandry complied with the ARRIVE guidelines (Kilkenny et al., 2010). The ethical procedures and policies were approved by the Research Ethics Committee of the Faculty of Medicine for Girls, Al-Azhar University (FMG-IRB, approval no. RHBIRB2018122001), Cairo, Egypt. All efforts were made to minimize animal suffering and to reduce the number of animals used.

### 2.2 Materials

The FLE, secoisolariciresinol diglucoside (SDG) (CAS No. 148244-82-0, product No. S0202), and the apelin-13 receptor blocker, F13A (CAS No. 568565-11-7, product No. SML 2083), were purchased from Sigma-Aldrich Chemical Co. (St. Louis, MO, United States). FLE was dissolved in normal saline (5 mg/mL) and administered by oral gavage at the dose of 20 mg/kg (Pilar et al., 2017), while F13A was dissolved in normal saline to prepare a stock solution of 100 μg/mL (Soltani Hekmat et al., 2011). The commercial rat chow diet (balanced diet) was composed of 23.5% protein, 48.8% carbohydrate, 5% lipid, 12% water, 5% ash, 5% cellulose, and a 0.7% mixture of vitamins and minerals which provided a total of 310 kcal/100 g diet. This diet was designed at the National Research Institute for Nutrition, Giza, Egypt, according to the National Nutrition Database for Standard Reference. Diet was purchased from EL Nasr Pharmaceutical Chem. Co., New Maadi, Cairo, Egypt.

The CAFD is also known as the Junk food or high-calorie fast food diet which is a well-established model to study obesity; it is a highly palatable hyperlipidemic diet that mimics the Western diet, inducing rapid weight gain in rodents (Johnson et al., 2016; Sabater et al., 2016). The CAFD was composed of beef burgers, bread, and mayonnaise. Each piece of beef burger weighed 150 g before cooking and loses between 15 and 20 g after cooking, resulting in an average new weight of 130–135 g/piece. Each 100 g of the uncooked beef burger was composed of 18% protein, 17.7% total fat, 6.12% total saturated fatty acid, 45% carbohydrates, 12.3% water, and 0.8% vitamin mixture, providing 330 kcal according to the nutritive values recorded on the package. As for mayonnaise, each tablespoonful has 97 calories and is composed of 11 g fat, 1.5 g saturated fat, 10 mg cholesterol, and 85 mg sodium, according to the nutritive values recorded on the package. The CAFD provided a total of 415 kcal/100 g diet.

### 2.3 Experimental design

#### 2.3.1 Laboratory animals’ grouping and treatments

Thirty adult male Wistar rats weighing 150–170 g were purchased from the Nile Pharmaceuticals Company (Cairo, Egypt). They were housed in laboratory standard polypropylene cages (4 rats per cage), under specific pathogen-free conditions in facilities maintained at controlled room temperature (22°C ± 2°C) and relative humidity (55% ± 5%) with a natural 12-h light/12-h dark cycle. All animals had free access to a chow diet and water *ad libitum* and were acclimatized for 1 week before the initiation of the experiment.

The rats were divided into 5 equal groups (6 rats/group) as follows:

Group I (Control): rats were fed a standard diet and injected normal saline (1 mL/kg/day; iv) around 1:00 p.m. every day, 1 h before the administration of normal saline by oral gavage (10 mL/kg/day; po) (≈2:00 p.m.) for 12 weeks.

Groups II (FLE): rats were fed a standard diet and injected normal saline (1 mL/kg/day; iv) around 1:00 p.m. every day, 1 h before the administration of FLE (20 mg/kg/day) (Pilar et al., 2017) by oral gavage (≈2:00 p.m.) for 12 weeks.

Groups III (CAFD): rats were fed CAFD in addition to the standard chow for 12 weeks. Each rat was given one piece of beef burger fried in 15 g of sunflower oil, one tablespoonful of mayonnaise, and one piece of bun bread, weighing 60 g/piece (Abd Elwahab et al., 2017). To mimic the other groups’ conditions, rats were injected with normal saline (1 mL/kg/day; iv) around 1:00 p.m. every day, 1 h before the administration of normal saline by oral gavage (10 mL/kg/day; po) (≈2:00 p.m.) for 12 weeks.

Group IV (CAFD/FLE): In addition to the CAFD and standard chow as group III, rats were injected with normal saline (1 mL/kg/day; iv) around 1:00 p.m. every day, 1 h before the administration of FLE (20 mg/kg/day) by oral gavage (≈2:00 p.m.) for 12 weeks.

Group V (CAFD/FLE/F13A): In addition to the CAFD and standard chow, rats were injected with the apelin-13 antagonist (F13A) (30 μg/kg/day; iv) (Najafipour et al., 2012) around 1:00 p.m. every day, 1 h before the administration of FLE (20 mg/kg/day) by oral gavage (≈2:00 p.m.) for 12 weeks.

The standard chow and the prepared CAFD were introduced for individually-caged rats. The standard chow and the CAFD were weighed before consumption to introduce the same amount of diet/rat (25 g/day). The daily food and calorie consumptions were calculated by subtracting the amount of leftover, after 24 h, from the amount of food initially given. Both, the standard and CAFD were introduced daily at 10 a.m.

All treatments were given at the same time every day. For the iv injection of F13A, a catheter was placed in the lateral caudal vein of the tail. The catheters were flushed daily with 0.2 mL of heparinized 0.9% saline to maintain patency and once/week with 0.2 mL gentamycin (10 mg/mL) to prevent infection.

#### 2.3.2 Measurement of body weight, food consumption, and caloric intake

Food and caloric intake were measured daily, while weight gain was monitored weekly for each rat at approximately the same time for 12 weeks.

#### 2.3.3 Blood sampling and tissue preparation

At the end of the experiment, all rats were anesthetized with a cocktail of i.p. ketamine/xylazine (87/13 mg/kg) injected at the dose of 1 mL/kg (Ala et al., 2021), then blood samples were collected from retro-orbital sinus and left to clot for 1 h at room temperature, then centrifuged at 3,000 rpm for 15 min to separate sera. Subsequently, all rats were sacrificed by decapitation and the heart was dissected and divided into two portions. The first portion was immersed immediately in 10% neutral buffered formalin and processed for histopathological examination. The second portion was sliced into small pieces and then stored at −80°C until biochemical investigations. All experiments were performed under blind conditions.

### 2.4 Methods

#### 2.4.1 Assessment of serum lipid profile

Serum TC, high-density lipoprotein-cholesterol (HDL-C), and triglycerides (TG) were measured by spectrophotometric methods using the corresponding kits from Biodiagnostic (Egypt). As for LDL-C, it was calculated by the formula: LDL-C = $TC-HDL-C-\left(TG/5\right)$ , according to Friedewald et al. (1972). The oxidized LDL (oxLDL) was assessed in serum using a relevant sandwich ELISA kit from MyBioSource (San Diego, CA, United States, catalog # MBS262297).

#### 2.4.2 Atherogenic indices

Expressing imbalances between atherogenic and cardioprotective lipoproteins, such indexes are powerful indicators of CVD risk and could be useful for evaluating response to therapeutic intervention. They were computed according to the following formulas.

Atherogenic index (AI) = log $\left(TG/HDL-C\right)$ (Ikewuchi et al., 2011).

Atherogenic coefficient (AC) = $\left(TC-HDL-C\right)/HDL-C$ (Ikewuchi et al., 2013).

Cardiac risk ratio (CRR) = TC/HDL−C (Kinosian et al., 1994).

#### 2.4.3 Assessment of serum oxidative stress markers and inflammatory cytokines

Malondialdehyde (MDA), the end product of lipid peroxidation, was measured following the method of Ohkawa et al. (1979). Reduced glutathione (GSH) was assessed according to the method of Tietze (1969). Tumor necrosis factor alpha (TNF-α) and interleukin-6 (IL-6) serum levels were measured by their respective ELISA kits (RayBiotech, Peachtree Corners, GA, United States) according to the manufacturer’s protocol.

#### 2.4.4 Assessment of serum markers of endothelial function

Nitric oxide (NO) level was measured by using a commercial kit (Catalog # 2533) from Biodiagnostic, Cairo, Egypt, according to the manufacturer’s instructions. Endothelin-1 (ET-1) was determined using an ET-1 ELISA kit (Kamiya Biomedical Company, Tukwila, WA, United States, catalog # KT-14033). Vascular cell adhesion molecule-1 (VCAM1) was determined by the ELISA kit of MyBioSource (San Diego, CA, United States, catalog # MBS726819). Serum paraoxonase 1 (PON1) activity was determined spectrophotometrically using paraoxon (O,O-diethyl-O-P nitrophenylphosphate) as the substrate purchased from Sigma-Aldrich (St. Louis, MO, United States). The initial velocity of phenol formation during the hydrolysis of phenyl acetate was calculated from the increase of absorbance at 270 nm.

#### 2.4.5 Assessment of markers of cardiac injury and thrombotic activity

Serum C-reactive protein (CRP) level was measured by an ELISA kit (MyBioSource, San Diego, CA, United States; catalog # MBS2508830). Cardiac troponin I (cTnI) was quantitatively measured in serum using an ELISA kit (Elabscience^®^, Houston, TX, United States). Serum plasminogen activator inhibitor-1 (PAI-1) was determined by its respective ELISA kit, Hyphen BioMed, Paris, France.

#### 2.4.6 Evaluation of cytokine production in cardiac tissue homogenate

The production of TNF-α, interferon-gamma (IFN-γ), and IL-10 were evaluated in cardiac homogenate, using quantitative ELISA commercial kits (R & D Systems, Minneapolis, MN, United States) according to the manufacturer’s instructions.

#### 2.4.7 Estimation of cardiac apoptotic markers

The activity of myocardial caspase-3 was determined using a commercial colorimetric kit (Sigma-Aldrich, St. Louis, MO, United States) as per the standard methods provided. Caspase-3 activity was expressed in nmol pNA/min/mL. Bcl2-associated X (Bax) protein level was measured according to the manufacturer’s instructions for commercial kits (CUSABIO, Wuhan, China) through an ELISA-type method.

#### 2.4.8 Real-time quantitative polymerase chain reaction (RT-qPCR)

Total RNA was extracted from cardiac tissue homogenate using RNeasy Kit (Qiagen, Hilden, Germany), and the tissue lysate was centrifuged for 3 min at 10,000 x g then the supernatant was taken. The supernatant was reverse transcribed into cDNA using a High-Capacity cDNA Reverse Transcription Kit (Applied Biosystems™, Foster City, CA, United States) according to the manufacturer’s guidelines. To assess the expression of target genes, qPCR was performed using SYBR^®^ Green PCR Master Mix (Applied Biosystems™, Foster City, CA, United States) as described by the manufacturer. The relative expression of target genes was obtained using the ΔΔ CT method as previously described by Schmittgen and Livak (2008) using β-actin as a housekeeping gene (Table 1).

**TABLE 1:** Primer sequences used for real-time quantitative polymerase chain reaction (RT-qPCR).

Gene	GenBank accession number	Forward primer	Reverse primer
Nrf2	**NM_031789**	TTG​TAG​ATG​ACC​ATG​AGT​CGC	ACT​TCC​AGG​GGC​ACT​GTC​TA
MMP-9	**NM_031055.1**	ACG​GCA​AGG​ATG​GTC​TAC​TG	AGT​TGC​CCC​CAG​TTA​CAG​TG
FOXO3a	**NM_001106395**	GTC​CCT​GAA​GGG​AAG​GAG​C	CTC​GTC​CAG​GAT​GGC​GTA​G
Apelin-13	**NM_031612.3**	TGG​AAG​GGA​GTA​CAG​GGA​TG	TCCTTATGCCCACT
APJ	**NM_031349.2**	GGA​CTC​CGA​ATT​CCC​TTC​TC	CTT​GTG​CAA​GGT​CAA​CCT​CA
β-actin	**NM_031144**	GGT​CGG​TGT​GAA​CGG​ATT​TGG	ATG​TAG​GCC​ATG​AGG​TCC​ACC

#### 2.4.9 Extraction of cytosolic and nuclear fraction

Cytosolic and nuclear fractions were separated from the cardiac tissue homogenate using ReadyPrep™ Protein Extraction Kit (Cytoplasmic/Nuclear) (Bio-Rad Laboratories, Irvine, CA, United States, catalog # 163-2089). In brief, 0.75 mL of cytoplasmic protein extraction buffer and protease inhibitor were added to 50 mg of cardiac tissue samples in the chilled Dounce homogenizer and then tissues were broken up by stroking. The Dounce homogenizer containing the homogenate was incubated on ice for 2 min. Afterward, the supernatant containing the cell lysate was transferred into a clean tube. The cell lysate was centrifuged at 1,000 x g for 10 min at 4°C. After centrifugation was completed, the supernatant that contained the cytoplasmic protein was transferred to a new tube, and the pellet that contained the nuclei was washed using cytoplasmic protein extraction buffer. Subsequently, the pellet was vortexed quickly to re-suspend the nuclei and was incubated on ice for 10 min. Finally, it was centrifuged to concentrate the nuclei.

#### 2.4.10 Immunoblotting

Protein concentration in cardiac tissue samples was determined using a Bradford protein assay kit according to the manufacturer’s protocol (Bio-Rad Laboratories, Irvine, CA, United States, catalog # 500-0201). Equal protein concentration samples (≈20 μg) were loaded on 10% Sodium Dodecyl Sulfate-polyacrylamide gel (SDS-PAGE) and separated by electrophoresis before being transferred to Immun-Blot^®^ polyvinylidene difluoride membranes (Bio-Rad Laboratories, Irvine, CA, United States). Next, the membranes were blocked with 5% (w/v) skimmed milk powder in Tris Buffered Saline-Tween (TBS-T) for 2 h at room temperature. The membranes were incubated overnight with the primary antibodies against apelin-13 (1:2000) [Thermo Fisher Scientific, Waltham, MA, United States, catalog # PA5-114860], APJ (1:1000) [Thermo Fisher Scientific, Waltham, MA, United States, catalog # PA5-114830], p-AMPK alpha (Thr172) (1:2000) [Biorbyt Ltd., Cambridge, UK, catalog # orb5692], AMPK alpha 2 (1:2000) [Biorbyt Ltd., Cambridge, UK, catalog # orb556094], p-FOXO3a (Ser574) (1:1000) [Biorbyt Ltd., Cambridge, UK, catalog # orb1629858], FOXO3a (1:1000) [Biorbyt Ltd., Cambridge, UK, catalog # orb385614], Nrf2 (1:2000) [Thermo Fisher Scientific, Waltham, MA, United States, catalog # PA5-88084], β-actin (1:1000) (Santa Cruz Biotechnology, Santa Cruz, CA, United States, catalog # sc-47778), and histone H3 (1:1000) (Santa Cruz Biotechnology, Santa Cruz, CA, United States, catalog # sc-517576). The blots were then washed three times with TBS-T. Afterward, the blots were incubated with anti-rabbit horseradish peroxidase-conjugated secondary antibody (1:10000) (Bio-Rad Laboratories, Irvine, CA, United States, catalog # 170–6515) for 1 h at room temperature followed by washing with TBS-T three times. Finally, the membranes were visualized using SuperSignal™ West Femto chemiluminescent substrate (Thermo Fisher Scientific, Waltham, MA, United States), and densitometrical quantification was conducted using ImageJ software (Bio-Rad Laboratories, Irvine, CA, United States). The bands’ intensities were normalized to the intensity of the corresponding total protein, β-actin (for cytosolic enriched fraction), or histone H3 (for nuclei enriched fraction).

### 2.5 Histological examination

The heart was carefully dissected to take the cardiac muscles from the lower one-third of the left ventricle, which was immediately fixed in 10% neutral buffered formalin at room temperature. The tissues were dehydrated using ascending grades of alcohol, cleared in xylene, and embedded in paraffin, and then sections were cut at 5–7 μm with a rotatory microtome and mounted on glass slides. The sections were stained with hematoxylin and eosin (H&E) for routine histological examination and Masson’s trichrome stain for the detection of collagen fibers (Bancroft and Gamble, 2008). Periodic acid–Schiff (PAS) stain was used for the detection of glycogen content in cardiac muscle (Singh, 2003).

### 2.6 Morphometric examination

The percentage of areas of collagen was measured morphometrically in the wall of heart muscles in Masson’s trichrome-stained sections via a computerized image system composed of a Leica Quin 500 image analyzer, connected to a Leica microscope. The optical density (O.D.) of PAS-stained sections was measured using the NIH Image J (v1.50) program. Optical density was calculated by the following formula:

Optical density = log $\left.\left(maximum\;intensity\right./mean\;intensity\right)$

Where maximum intensity = 255 for 8–bit images.

### 2.7 Statistical analysis

Each variable was assessed for normality using the Kolmogorov-Smirnov test. All data were normally distributed, thus, followed parametric analysis and were expressed as means ± SD and compared using one-way ANOVA followed by Tukey’s *post hoc* test. Repeated measures ANOVA followed by Tukey’s *post hoc* test was used for comparisons of body weight, food consumption, and caloric intake changes among groups over the 12 weeks. A *p-*value < 0.05 was considered statistically significant. Statistical analysis was performed using GraphPad Prism^®^, version 9.1.0, a statistical software program (GraphPad Software, Inc., San Diego, CA, United States, RRID: *SCR_002798*).

## 3 Results

The statistical comparisons between the control and FLE (20 mg/kg/day) groups revealed no significant differences; therefore, all comparisons refer to the control group.

Effect of FLE on body weight, food consumption, caloric intake, lipid profile, and atherogenic indices in CAFD-fed rats.

**FIGURE 1:**
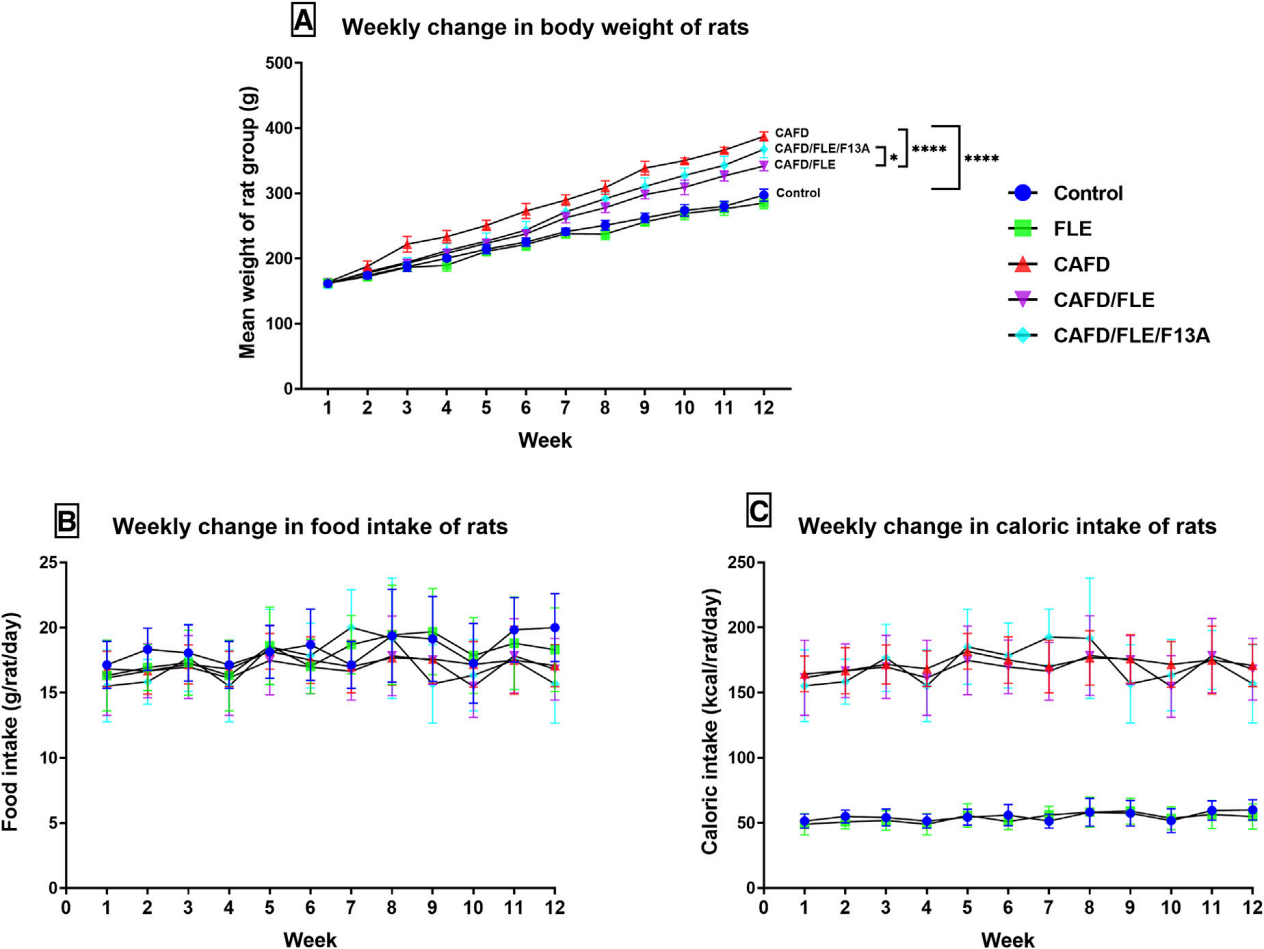
The mean change of the **(A)** body weight, **(B)** food intake, and **(C)** caloric intake per week in the control, cafeteria diet (CAFD)-, flaxseed lignan extract (FLE)-, and FLE/F13A-treated rat groups over 12 weeks. F13A is an apelin-13 receptor blocker. Values are presented as the mean ± SD (*n* = 6 per group; repeated measures ANOVA followed by Tukey’s multiple comparison test; ^*^ *p* < 0.05, ^****^ *p* < 0.0001).

As shown in Figure 1A all groups showed similar initial body weights. However, after 12 weeks all groups showed significantly higher final body weight when compared to their initial one.

The CAFD-fed rats started to reveal a significant increase in their body weights at week 2 (*p* = 0.0335) to reach a final body weight significantly higher by 30.3% when compared to the control group (Figure 1A; Table 2). The CAFD/FLE-treated rats started to show a significant difference in their body weights at week 3 (*p* = 0.0057) to reach a significantly lower final weight by 11.8% when compared to the CAFD-fed group by the end of the 12^th^ week. Administration of the APJ blocker, F13A, reduced the effect of FLE, where the final body weight of the CAFD/FLE/F13A group was significantly higher by 7.5% when compared to the CAFD/FLE-treated rats (*p* = 0.0194).

**TABLE 2:** Effect of flaxseed lignan extract (FLE) on final body weight, food intake, serum lipid profile, and atherogenic indices in experimental rat groups.

	Control	FLE	CAFD	CAFD/FLE	CAFD/FLE/F13A
Final body weight (g)	297.2 ± 9.3	285.0 ± 9.1	387.4 ± 6.9^****^	341.8 ± 7.3^####^	367.5 ± 13.2^@@@^
Food Intake (g/day)	18.4 ± 1.2	17.9 ± 1.6	17.3 ± 1.1	16.9 ± 2.0	17.1 ± 1.5
Serum TC (mg/dL)	86.8 ± 8.6	84.6 ± 8.7	195.2 ± 7.7^***^	88.3 ± 6.4^###^	180.1 ± 4.7^@@@^
Serum TG (mg/dL)	63.3 ± 6.2	60.2 ± 6.5	128.2 ± 6.3^***^	66.4 ± 6.7^###^	77.6 ± 3.8^@^
Serum LDL-C (mg/dL)	32.1 ± 10.3	26.8 ± 11.6	148.9 ± 8^***^	33 ± 9.9^###^	127.2 ± 3.5^@@@^
Serum HDL-C (mg/dL)	42.1 ± 6.5	45.8 ± 7	20.7 ± 4.2^***^	42 ± 7.6^###^	34.6 ± 1.8
Serum oxLDL (ng/mL)	5.9 ± 0.6	5.6 ± 0.7	11.1 ± 0.9^****^	8.6 ± 1^##^	10.4 ± 1.5^@^
AI	0.18 ± 0.07	0.12 ± 0.1	0.8 ± 0.1^***^	0.20 ± 0.09^###^	0.35 ± 0.03^@^
AC	1.1 ± 0.36	0.9 ± 0.46	8.8 ± 2.2^***^	1.2 ± 0.49^###^	4.2 ± 0.28^@@@^
CRR	2.1 ± 0.36	1.9 ± 0.46	9.8 ± 2.2^***^	2.2 ± 0.49^###^	5.2 ± 0.28^@@@^

Data are presented as the mean ± SD (*n* = 6 per group); one-way ANOVA followed by Tukey’s multiple comparison test.

**p < 0.01, ***p < 0.001, vs. the control group.

^###^ *p* < 0.001, vs. the CAFD-treated group.

^@^ *p* < 0.05, ^@@@^ *p* < 0.001 vs. CAFD/FLE-treated group.

AC, atherogenic coefficient; AI, atherogenic index; CRR, cardiac risk ratio; HDL-C, high-density lipoprotein-cholesterol; LDL-C, low-density lipoprotein-cholesterol; oxLDL, oxidized low-density lipoprotein; TC, total cholesterol; TG, triglycerides.

The mean food consumption of each group showed a slight increase by the end of week 12 (Figure 1B), however, this increase was non-significant in all groups. Moreover, the average daily food intake was non-significant in all groups (Table 2).

The caloric intake of standard chow-fed rats did not show any significant difference over the 12 weeks experimental period (Figure 1C). Similarly, the groups fed CAFD showed a non-significant change in caloric intake during the whole experimental period. However, the caloric intake of groups fed standard chow was significantly lower than those fed CAFD (*p* < 0.0001).

Moreover, the assessed lipid profile in Table 2 showed overt dyslipidemia after CAFD for 12 weeks, represented by significant increases in TC (2.2-fold), TG (2-fold), LDL-C (4.6-fold), and oxLDL (1.9-fold) concurrently with a 50.8% decrease in HDL-C, when compared to the control group. These alterations were returned near the corresponding control levels after FLE consumption for 12 weeks, showing a significant decrease in TC, TG, LDL-C, and oxLDL by 54.8%, 48.2%, 77.8%, and 22%, respectively, along with a 2-fold increase in HDL-C, as compared to CAFD-treated animals. Concurrent administration of F13A reduced the effect of FLE on lipid profile, showing significantly higher TC (2-fold), TG (16.9%), LDL-C (3.9-fold), and oxLDL (20.6%) except for HDL-C which was not significantly different when compared to CAFD/FLE group.

As for the listed atherogenic indices in Table 2, there was a significant rise in AI (4.4-fold), AC (8-fold), and CRR (4.7-fold) in the CAFD group, when compared to the control. Treatment with FLE significantly ameliorated these elevations approaching the control pattern, where AI, AC, and CRR were significantly lower by 75%, 86.4%, and 77.6%, respectively, as compared to the CAFD group. Administration of F13A reduced the latter effect of FLE, where AI, AC, and CRR were significantly higher by 75%, 3.5-fold, and 2.4-fold, respectively, when compared to the CAFD/FLE group.

### 3.1 Effect of FLE on oxidative stress and inflammation markers in CAFD-fed rats

**FIGURE 2:**
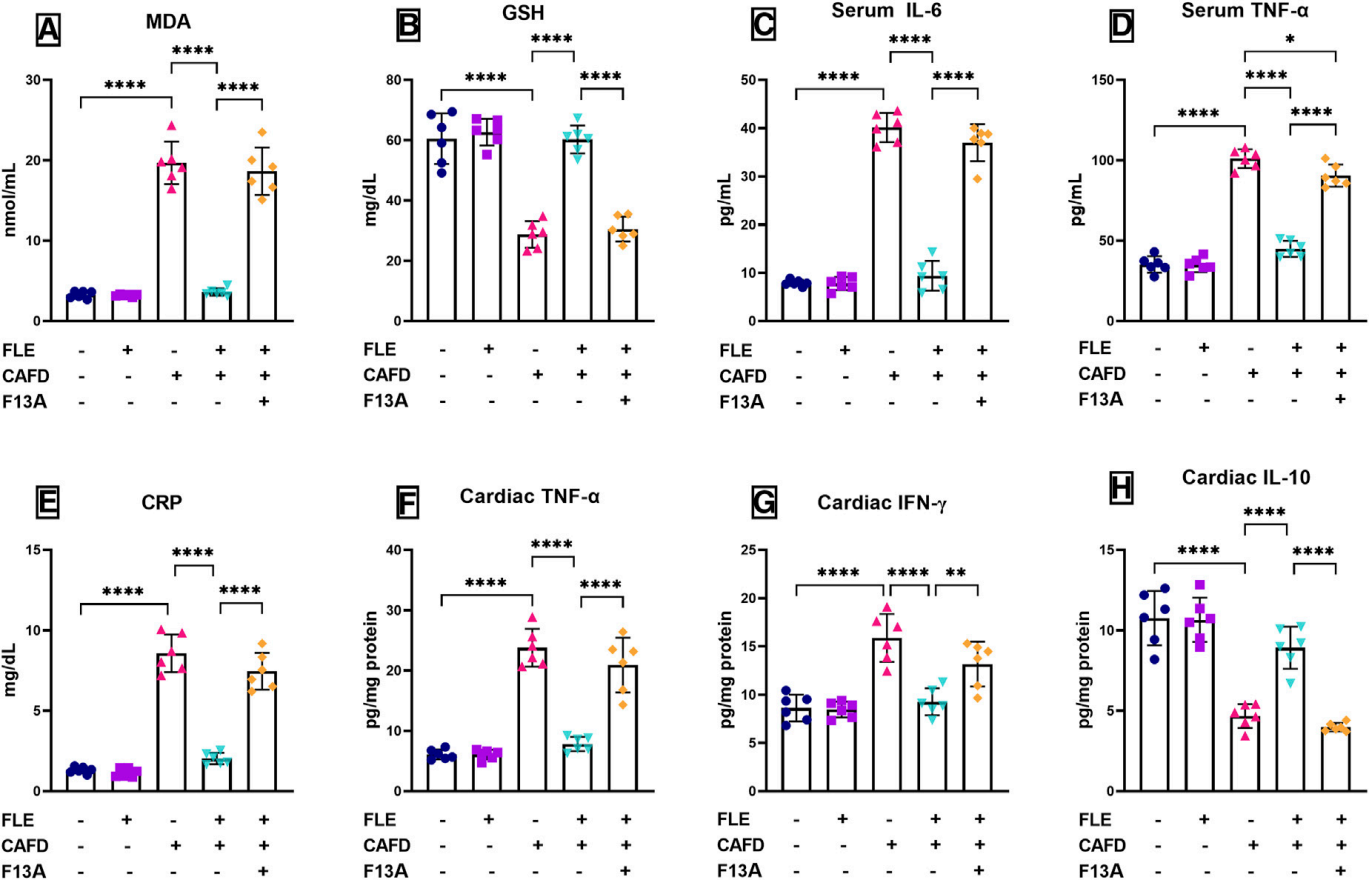
Effect of flaxseed lignan extract (FLE) on serum **(A)** malondialdehyde (MDA), **(B)** reduced glutathione (GSH), **(C)** interleukin-6 (IL-6), **(D)** tumor necrosis factor-alpha (TNF-α), and **(E)** C-reactive protein (CRP), as well as the cardiac tissue levels of **(F)** TNF-α, **(G)** interferon-gamma (IFN-γ), and **(H)** IL-10 of the cafeteria diet (CAFD)-fed rats. F13A is an apelin-13 receptor blocker. Values are presented as the mean ± SD (*n* = 6 per group; one-way ANOVA followed by Tukey’s multiple comparison test; ^*^ *p* < 0.05, ^**^ *p* < 0.01, ^****^ *p* < 0.0001).

Oxidative stress was induced by CAFD, presented by the significant increase of serum MDA (6-fold) and 52.4% significant reduction of GSH, as compared to the control group (Figures 2A, B). Similarly, serum inflammatory markers were significantly elevated in CAFD-treated rats as follows; IL-6 (5.1-fold), TNF-α (2.9-fold), and CRP (6.5-fold), as compared to their control levels (Figures 2C–E). In the examined cardiac tissues, CAFD-treated rats showed a significant increase in the levels of TNF-α and IFN-γ by 3.9- and 1.8-fold, respectively (Figures 2F, G). However, the anti-inflammatory IL-10 levels were significantly decreased by 56.6% when compared to the control ones (Figure 2H). These levels were successfully corrected by FLE consumption, displaying significantly reduced MDA, IL-6, TNF-α, and CRP by 81.6%, 76.5%, 55.5%, and 76.4%, respectively, along with significant elevation in GSH by 2.1-fold, as compared to CAFD group**.** Similarly, markers assessed in the cardiac tissues were successfully improved by FLE consumption, as revealed in the significant reduction of TNF-α and IFN-γ levels by 67.1% and 41.7%, respectively, together with significant elevation in IL-10 levels by 90.8%, as compared to the CAFD group.

Simultaneous administration of APJ blocker, F13A, lowered the correcting effect of FLE, showing 5.2-, 3.9-, 2-, and 3.7-fold significant increases in MDA, IL-6, TNF-α, and CRP, respectively, along with 49.3% significant decrease in GSH, when compared to CAFD/FLE group. In alignment, the co-administration of F13A with FLE showed significantly higher cardiac values as follows; TNF-α (2.7-fold) and IFN-γ (42.3%), along with 55.4% significant reduction in IL-10 levels when compared to the CAFD/FLE group.

### 3.2 Effect of FLE on CAFD-induced endothelial dysfunction, cardiac injury markers, and thrombotic activity

**FIGURE 3:**
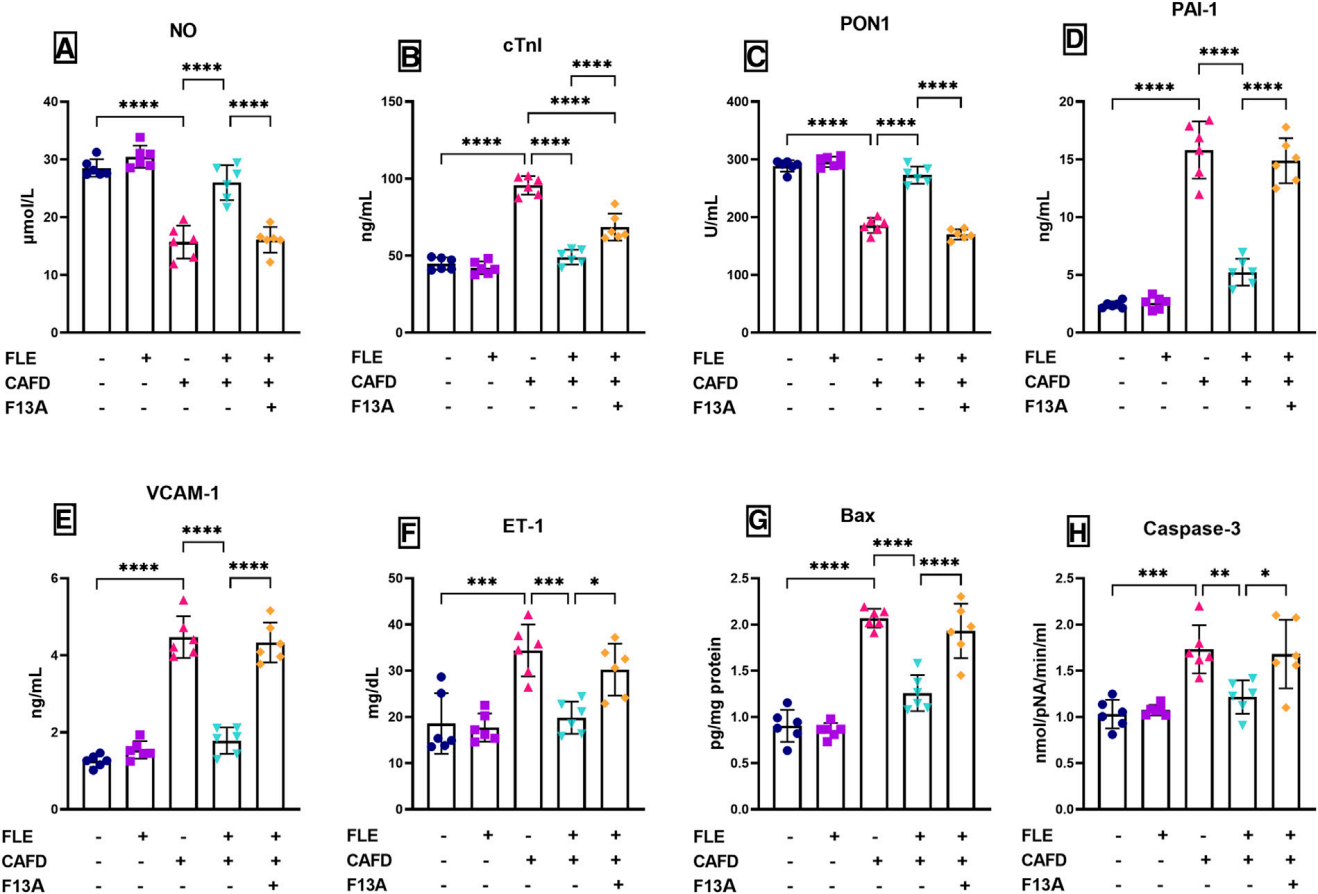
Effect of flaxseed lignan extract (FLE) on the serum levels of **(A)** nitric oxide (NO), **(B)** cardiac troponin I (cTnI), **(C)** paraoxonase-1 (PON1), **(D)** plasminogen activator inhibitor-1 (PAI-1), **(E)** vascular cell adhesion molecule 1 (VCAM1), and **(F)** endothelin-1 (ET-1), as well as the **(G)** Bcl-2-associated X protein (Bax) levels, and **(H)** caspase-3 activity in the cardiac tissue of the cafeteria diet (CAFD)-fed rats. F13A is an apelin-13 receptor blocker. Values are presented as the mean ± SD (*n* = 6 per group; one-way ANOVA followed by Tukey’s multiple comparison test; ^*^ *p* < 0.05, ^**^ *p* < 0.01, ^***^ *p* < 0.001, ^****^ *p* < 0.0001).

As presented in Figures 3A–C, CAFD-treated rats showed a significant increase in CAFD-induced endothelial dysfunction, thrombotic activity, and cardiac injury, demonstrated by the 2.1-fold significant increase in serum cTnI, as well as a significant reduction in serum NO and PON1 by 44.9% and 35.6%, respectively, when compared to control values. Moreover, CAFD-treated rats showed increased serum levels of PAI-1, VCAM1, and ET-1 by 6.5-, 3.6-, and 1.9- fold, respectively, as compared to the control ones (Figures 3D–F).

These levels were successfully corrected by FLE approaching the control values, displaying significantly reduced cTnI, PAI-1, VCAM1, and ET-1 by 48.6%, 66.9%, 60.2%, and 42.4%, respectively, together with significant elevation in NO (65.6%) and PON1 (46.9%), as compared to CAFD group. Concurrent administration of F13A reversed the correcting effect of FLE, showing significantly higher serum levels as follows; cTnI (39.6%), PAI-1 (2.8-fold), VCAM1 (2.4-fold) and ET-1 (52.4%), along with 38.2% and 37.6% significant reduction in NO and PON1, respectively, when compared to CAFD/FLE group.

### 3.3 Effect of FLE on CAFD-induced apoptosis in cardiac tissues

The assessed apoptosis indicators (Bax levels and caspase-3 activity) in the cardiac tissues of the CAFD group showed 2.3- and 1.7-fold significant increases, as compared to the respective control values (Figures 3G, H). These changes were successfully improved by FLE consumption, as revealed by the significant reduction of cardiac Bax levels and caspase-3 activity by 39.2% and 29.8%, respectively, as compared to the CAFD group. Co-administration of the APJ antagonist (F13A) with FLE showed significant elevation in apoptosis markers as follows; Bax levels (53.5%) and caspase-3 activity (38.4%), when compared to the CAFD/FLE group.

### 3.4 Effect of FLE on gene expression of cardiac apelin-13, APJ, Nrf-2, MMP-9, and FOXO3a in CAFD-fed rats

**FIGURE 4:**
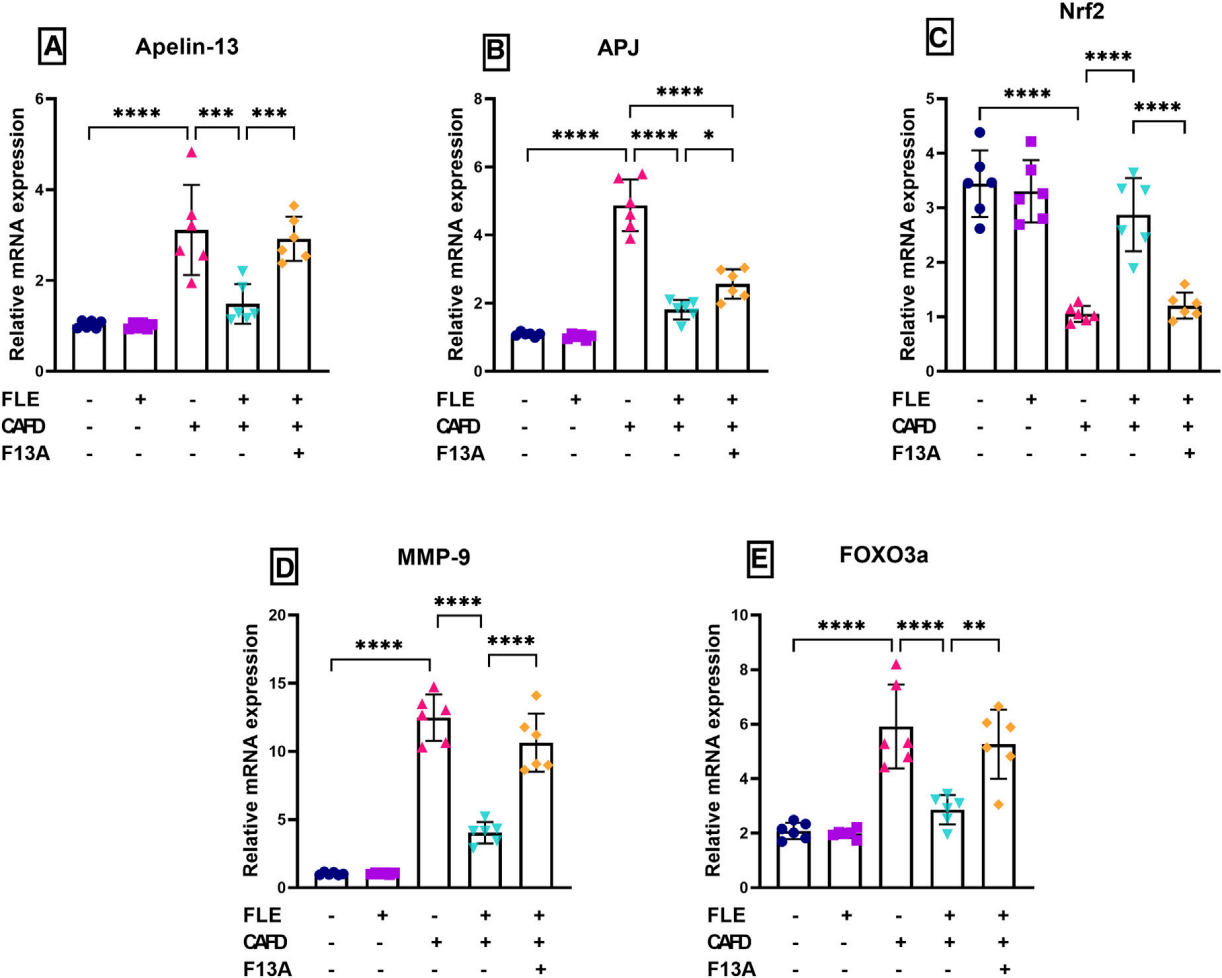
Effect of flaxseed lignan extract (FLE) on cardiac mRNA expression of **(A)** apelin-13, **(B)** apelin receptor (APJ), **(C)** nuclear factor erythroid 2-related factor 2 (Nrf2), **(D)** matrix metalloproteinase-9 (MMP-9), and **(E)** forkhead box O3 a (FOXO3a) in the cafeteria diet (CAFD)-fed rats. F13A is an apelin-13 receptor blocker. Values are presented as the mean ± SD (*n* = 6 per group; one-way ANOVA followed by Tukey’s multiple comparison test; ^*^ *p* < 0.05, ^**^ *p* < 0.01, ^***^ *p* < 0.001, ^****^ *p* < 0.0001).

As illustrated in Figures 4A, B, CAFD treatment resulted in a significant increase in gene expression of cardiac apelin-13 and its receptor, APJ, by 3- and 4.5-fold, respectively, as compared to their control levels. Moreover, increases in gene expression of cardiac MMP-9 (12.2-fold) and FOXO3a (2.8-fold), together with a 69.4% significant decrease in cardiac gene expression of Nrf2 were observed in CAFD-treated rats, as compared to its control levels (Figures 4C–E). Expression of these genes was ameliorated by FLE, revealed by the significantly reduced expression of apelin-13, APJ, MMP-9, and FOXO3a by 52.2%, 62.8%, 67.7%, and 51.6%, respectively, together with 2.7-fold significant elevation in Nrf2, as compared to CAFD group.

Concurrent administration of the APJ blocker, F13A, reduced the ameliorating effect of FLE, showing significantly higher cardiac expression of apelin-13 (2-fold), APJ (1.4-fold), MMP-9 (2.6-fold), and FOXO3a (1.8-fold), along with 58.1% significant reduction in Nrf2, when compared to CAFD/FLE group.

### 3.5 Effect of FLE on apelin-13, APJ, p-AMPK (Thr172), p-FOXO3a (Ser574), and Nrf2 (cytosolic/nuclear) protein expression in CAFD-fed rats

**FIGURE 5:**
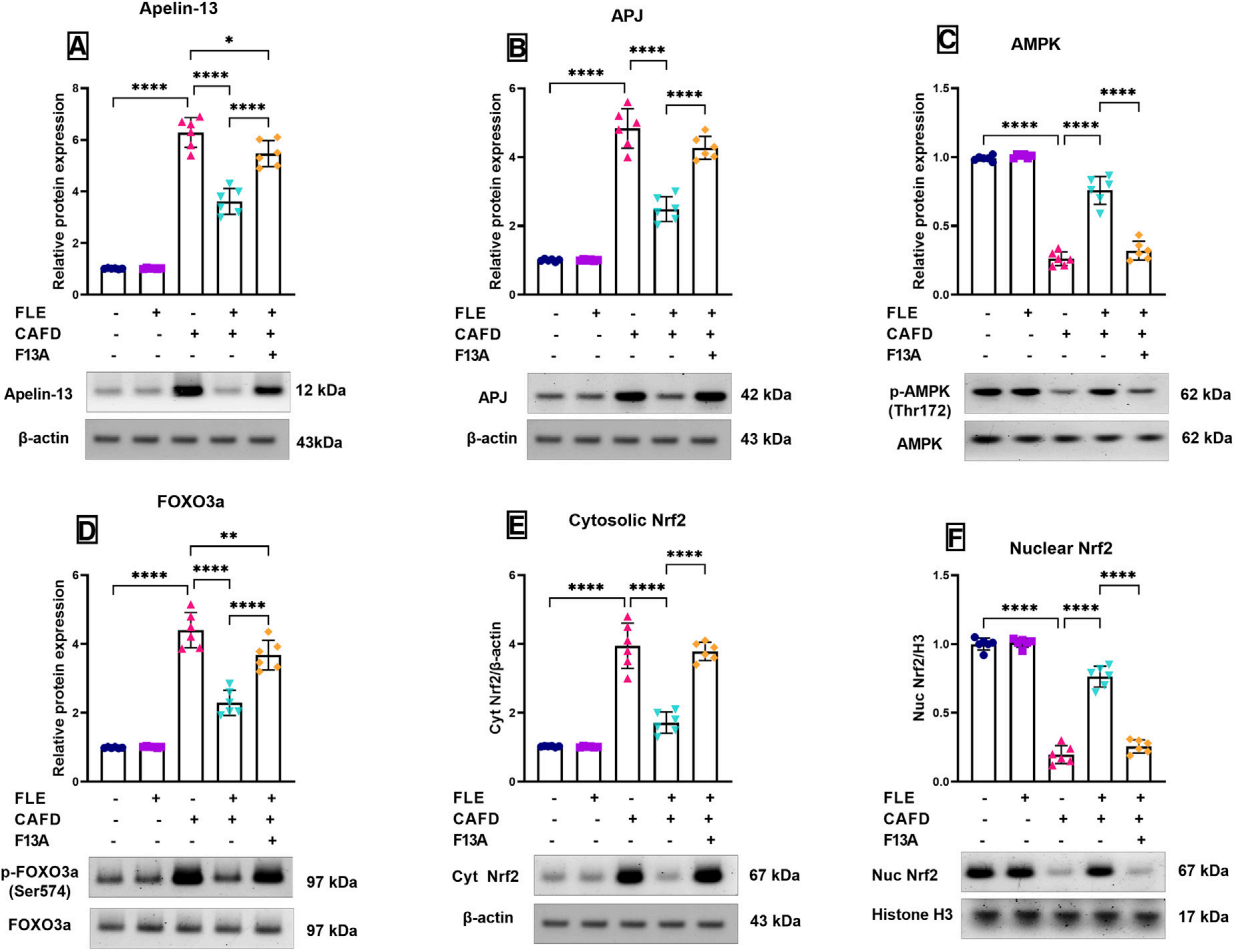
Relative protein expression levels and representative Western blot of **(A)** apelin-13, **(B)** apelin receptor (APJ), **(C)** phosphorylated AMP-activated protein kinase (p-AMPK) (Thr172), **(D)** phosphorylated forkhead box O3 a (p-FOXO3a) (Ser574), **(E)** cytosolic nuclear factor erythroid 2-related factor 2 (Nrf2), and **(F)** nuclear Nrf2 in the cardiac tissues of CAFD-fed rats treated with flaxseed lignan extract (FLE). Values are presented as the mean ± SD (*n* = 6 per group; one-way ANOVA followed by Tukey’s multiple comparison test; ^*^ *p* < 0.05, ^**^ *p* < 0.01, ^****^ *p* < 0.0001).

Figures 5A, B revealed a 6.2- and 4.8-fold increase in the protein expression levels of apelin-13 and its receptor, APJ, respectively, in the CAFD group when compared to the control group. Contrarywise, the CAFD/FLE treated group showed a significant decrease by 42.4% and 48.5% in the protein levels of apelin-13 and APJ, when compared to the CAFD group. The addition of the APJ blocker, F13A, led to a significant rise in the protein expression of apelin-13 and APJ by 51.2% and 71.6%, respectively, in comparison to the CAFD/FLE group.

As depicted in Figure 5C, the relative protein expression levels of the phosphorylated AMPK (Thr172) were significantly reduced by 73.5% in the CAFD group, when compared to the control group. However, FLE treatment in addition to the CAFD supplementation (CAFD/FLE group) managed to significantly increase the phospho-AMPK levels by 2.9-fold when compared to the CAFD alone. This effect was significantly reduced upon the addition of the blocker, F13A, where phospho-AMPK was significantly decreased in the CAFD/FLE/F13A group by 57.7% when compared to the CAFD/FLE group.

Inversely, the relative protein expression levels of the phosphorylated FOXO3a (Ser574) were significantly elevated by 4.4-fold in the CAFD group, when compared to the control group. Meanwhile, the supplementation of rats with FLE concomitantly with CAFD led to a significant reduction in the phospho-FOXO3a levels by 48% when compared to the sole intake of CAFD in rats. The addition of F13A (CAFED/FLE/F13A group) led to a significant rise in the phospho-FOXO3a levels by 60.5% when compared to the group receiving CAFD and FLE (Figure 5D).

In Figures 5E, F, the changes in the protein expression levels of both the cytosolic and nuclear fractions of Nrf2 are shown, respectively. The administration of CAFD showed a 3.8-fold significant increase in the cytosolic Nrf2 expression, together with an 80.3% reduction in the nuclear Nrf2 expression levels, which were statistically significant when compared to the control group. The treatment with FLE led to a significant nuclear translocation of Nrf2 as shown by the significant reduction of the cytosolic Nrf2 protein levels by 56.5% versus a significant elevation in the nuclear Nrf2 protein expression levels by 3.9-fold when compared to the CAFD group. The concurrent administration of the blocker, F13A, showed a negative impact on Nrf2 nuclear translocation, as there was a 2.2-fold significant increase in the cytosolic Nrf2 levels and a 66.4% significant reduction in the nuclear Nrf2 levels when compared to the group receiving FLE and CAFD.

### 3.6 Histological and morphometric results

**FIGURE 6:**
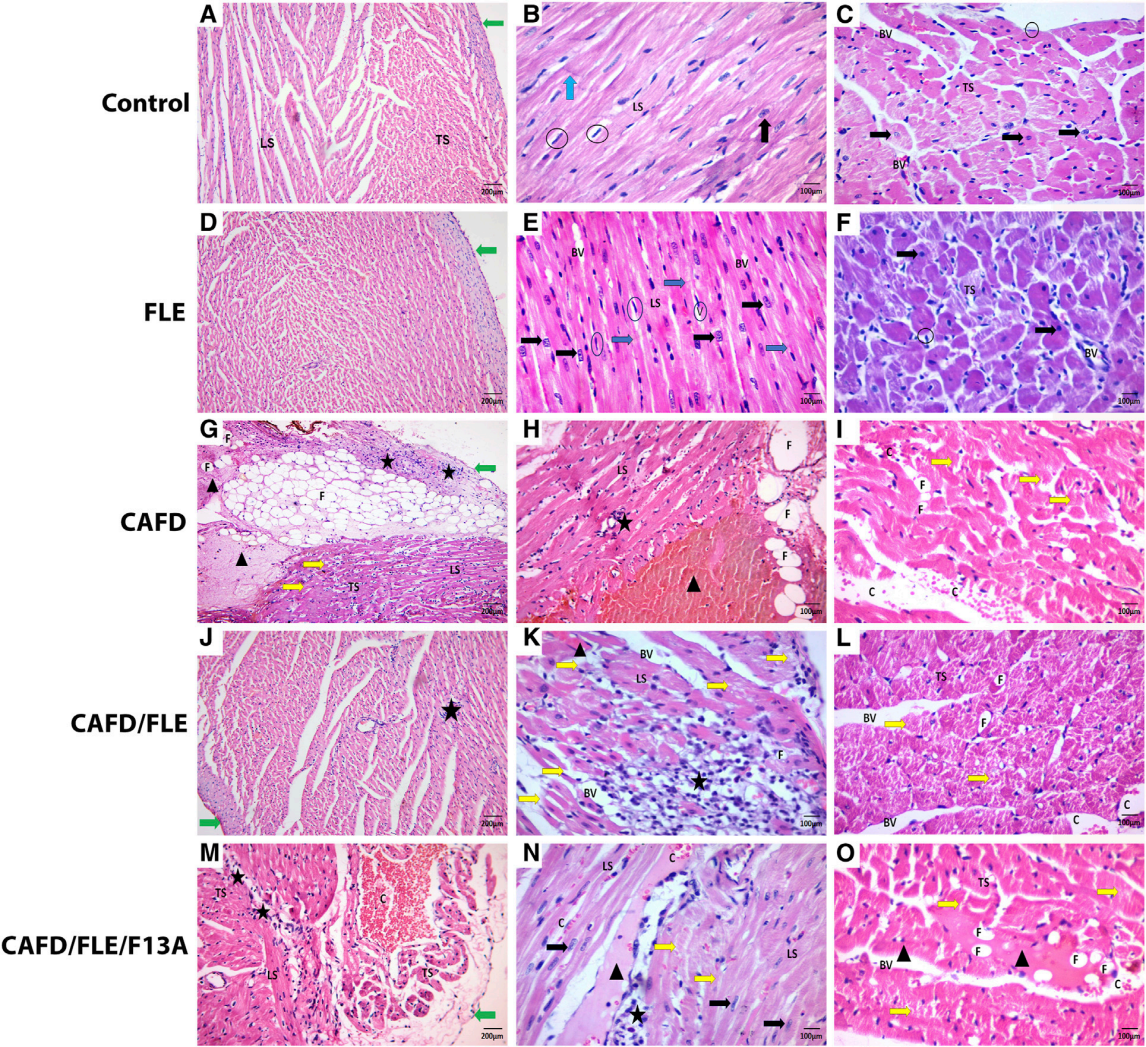
**(A,D)** The control and flaxseed lignan extract (FLE)-treated groups show irregularly arranged and anastomosing myocytes covered by perichondrium. **(B,E)** Longitudinal section (LS) of anastomosing muscle fibers with apparent striation and intercalated discs (Blue arrow), and central vesicular nuclei (Black arrow). **(C,F)** Transverse section (TS) of cardiac muscles having central vesicular nuclei and fibroblast nuclei (Circles). **(G–I)** The cafeteria diet (CAFD)-treated group shows thickened pericardium, with sub-pericardial hemorrhage and fat cells deposition (F) (LS & TS), separated cardiac myocytes with some destructed fibers (Yellow arrow) and degenerated areas (Arrowhead), fat cell infiltration (F), cellular infiltration (Star), and hemorrhage (C). **(J–L)** The CAFD/FLE-treated group shows pericardium and cardiac muscles (LS & TS) with less congestion (C), more cellular infiltrations (Star), and some destructed myocytes (Yellow arrow). **(M–O)** The CAFD/FLE/F13A-treated group shows irregularly arranged and widely separated muscle fibers (LS & TS), less hemorrhage (C), cellular infiltration (Star), as well as destructed (Yellow arrow) and degenerated myocytes (Arrowhead), where F13A is an apelin-13 receptor blocker. (H&E stain; X100, X200).

Light microscopic examination of H&E-stained sections from the control group revealed cardiac muscle surrounded by the simple squamous epithelium of pericardium. Cardiac myocytes appeared cylindrical, with branching and anastomosing fibers arranged in different directions. The muscle fibers had acidophilic cytoplasm with striation. They displayed single oval centrally located nuclei with visible nucleoli. Intercalated discs, which connect the muscle fibers, were detected. Each muscle fiber was surrounded by an endomysium of delicate connective tissue with fibroblast nuclei, which appeared flattened and darker stained than those of cardiac muscle cells (Figures 6A–C). The FLE-treated group showed the cardiac muscles more or less similar to the control one (Figures 6D–F).

Inversely, most of the cardiac muscle fibers of the CAFD-treated group had nearly normal myofibril structure but were widely separated by thickened endomysium with congestion and cellular infiltration by mononuclear inflammatory cells and fat cells. Some fibers were broken and discontinuous, and some showed degeneration with less obvious striation enclosing apoptotic nuclei. Parts of the cardiac muscles showed severe distraction and appeared as eosinophilic masses with no visible nuclei. Intercalated discs or transverse striation within these distracted fibers could not be seen. Thickened pericardium with sub-pericardial hemorrhage and infiltration by mononuclear cells and fat cells were detected (Figures 6G–I). These changes were ameliorated in cardiac sections of CAFD/FLE-treated rats (Figures 6J–L). However, sections from CAFD/FLE/F13A-treated group showed more distraction of the muscle with eosinophilic degeneration and mononuclear and fat cell infiltration. Furthermore, there was sub-pericardial hemorrhage and cellular infiltration (Figures 6M–O).

**FIGURE 7:**
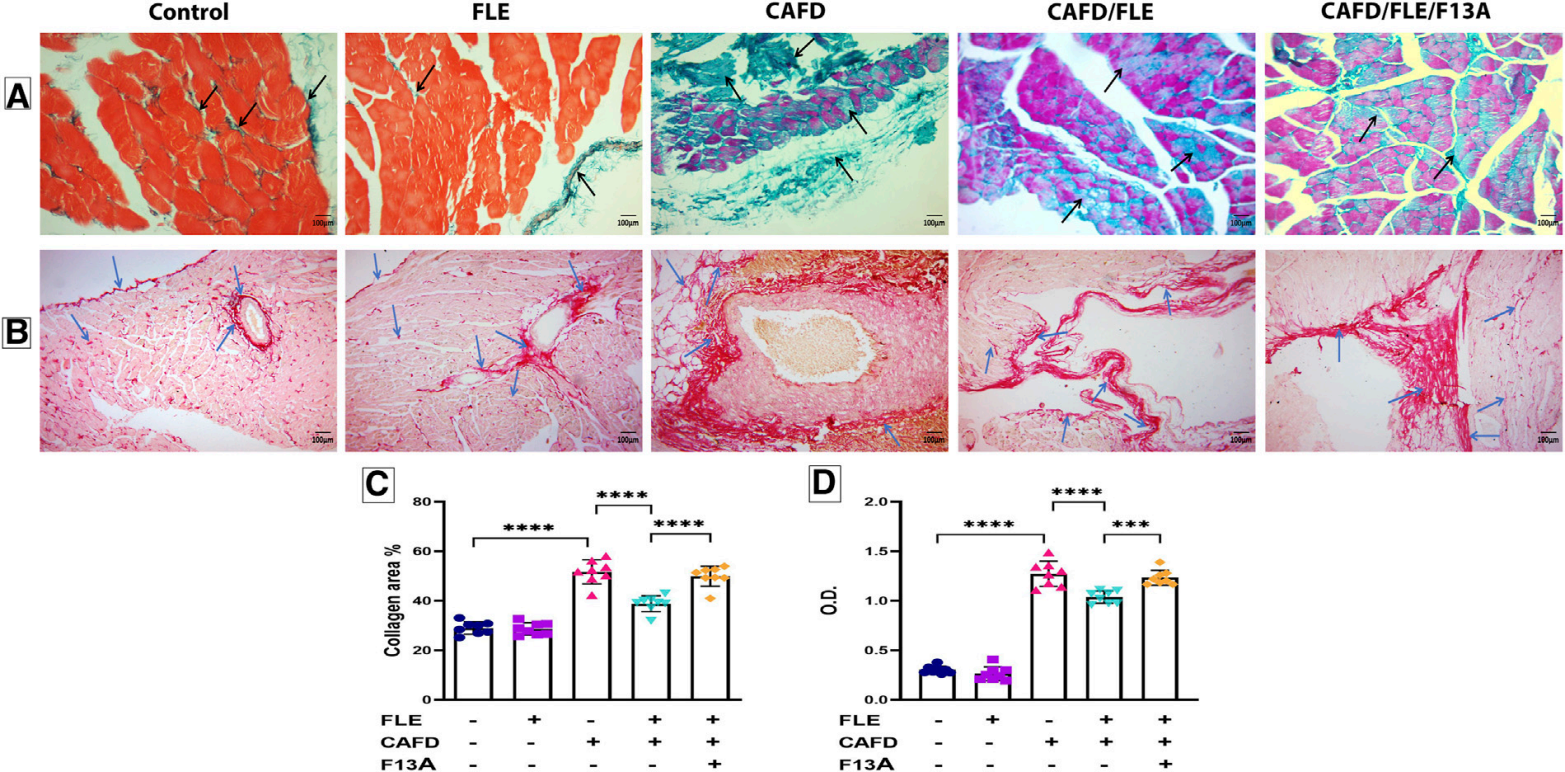
Photomicrographs of **(A)** Masson’s trichrome- and **(B)** Periodic acid–Schiff (PAS)-stained sections of cardiac muscles, **(C)** collagen area percentage, and **(D)** optical density (O.D.) of PAS reaction in rat groups. Masson’s trichrome and PAS; X200. Black arrows: collagen fibers. Blue arrows: positive reaction to the PAS stain. Data are presented as the mean ± SD (*n* = 6 per group; one-way ANOVA followed by Tukey’s multiple comparison test; ^***^ *p* < 0.001, ^****^ *p* < 0.0001). CAFD, cafeteria diet; FLE, flaxseed lignan extract, F13A; an apelin-13 receptor blocker.

As shown in Figures 7A, C, the examination of Masson trichrome-stained cardiac sections showed a slight amount of collagen fibers, mainly around blood vessels, in both the control and FLE-treated groups. There was a significant increase in the number of collagen fibers in between cardiac myocytes in the CAFD-treated group by 78.9%, as compared to the control group. The amount of collagen fibers was significantly decreased in CAFD/FLE-treated group by 25.5%, as compared to the CAFD-treated group. Nevertheless, a 29.2% significant increase in collagen fibers was observed in CAFD/FLE/F13A-treated group, in comparison to the CAFD/FLE-treated group.

Examination of the PAS-stained cardiac sections showed a positive PAS reaction in the pericardium, in between the muscle fibers and around blood vessels in the control and FLE-treated groups, which signified the glycogen content. PAS reaction was significantly higher in the CAFD-treated group as revealed by the 4.2-fold increase in O.D., in comparison to the control group. Noteworthy, decreased glycogen content was observed in CAF/FLE-treated group, as indicated by the 18.5% decrease in O.D. when compared to the CAFD-treated group. However, a 14% significant increase in the positive reaction of PAS stain was observed in CAFD/FLE/F13A-treated group, as compared to the CAFD/FLE-treated group (Figures 7B, D).

## 4 Discussion

The present work highlights the effectiveness of FLE in suppressing the pathogenic pathway of atherosclerosis and consequent vascular and cardiac injury that involves regulation of the apelin/APJ mechanistic pathway. To the best of our knowledge, we highlighted a novel cardioprotective effect of FLE in CAFD-induced cardiac fibrosis and vascular injury via modulation of apelin/APJ and its downstream signaling cascade axis in cardiac tissue (Supplementary Figure S1).

The evidence for the involvement of the apelinergic system in the pathogenic pathway of CAFD-induced cardiac fibrosis is the concomitant increase in APJ and apelin-13 gene expression. This came in agreement with a previous study (Czarzasta et al., 2016) whose authors suggested that the upregulation of the apelinergic system following exposure to a high-fat diet can have a positive function against oxidative stress. However, it is not yet clear whether this increase in apelin-13 levels during cardiovascular injury is correlated with protective or compensatory mechanisms of the apelinergic system (Mughal and O'Rourke, 2018). Inversely, this enhanced expression was reduced upon FLE treatment. Additionally, using the APJ blocker (F13A) was associated with increased expression of APJ and apelin-13. The impact of adding the APJ blocker, F13A, was shown in the reduced ameliorative effect of FLE on the involved indicators of inflammation, oxidative stress, and apoptosis of CAFD-induced cardiovascular pathogenesis, which implicates the beneficial role of apelin/APJ system (Lv et al., 2021).

The CAFD-fed group revealed increased levels of both serum and cardiac TNF-α, which may be an upstream mechanism involved in the increased mRNA expression of apelin (Ranjbar Kohan et al., 2018). The present study demonstrated that FLE treatment of CAFD-fed rats attenuated the increased apelin-13 and APJ gene expression in cardiac muscle. These changes were associated with reduced inflammatory cytokines and restored redox balance. The mechanism through which FLE downregulates apelin and APJ expression may be attributed to the weight reduction observed in CAFD-fed rats supplemented with FLE. This association was supported by a previous study, which reported that weight loss can lead to a significant reduction of tissue apelin expression and attributed it to the associated improved insulin sensitivity (Mehanna et al., 2018).

Supplementation of CAFD for 12 weeks, in the current work, resulted in cardiac fibrosis, oxidative stress, and apoptosis. This extent of cardiac fibrosis was evidenced by a significantly high percentage of collagen areas, and histologically by the significant distortions in the normal cardiomyocytes’ appearance and architecture. Some fibers showed a disarranged pattern with karyorrhexis of their nuclei, which may be caused by oxidative stress induced by the high-fat diet. There were signs of inflammation and fibrosis in interstitial and perivascular areas since recruited inflammatory cells are known to act on fibroblasts, which play a major role in the fibrosis process through the formation of pro-collagen (Sahraoui et al., 2016). Moreover, the myocardial accumulation of lipids might have contributed to the activation of the inflammatory processes and apoptosis (Frati et al., 2017). Inversely, the ameliorative effects of FLE were evidenced by decreased collagen percentage, and histologically by improved pericardium with normally separated cardiac muscles, and less separated anastomosing myocytes with less hemorrhage and cellular infiltrations.

In the current study, the rats in the CAFD group were found to crave the palatable higher caloric content CAFD more than the standard chow diet. Consequently, this led to a significant increase in the mean final body weight as well as overt dyslipidemia in the CAFD-fed rats. OxLDL is known to be a powerful inducer of endothelial injury via suppression of thrombomodulin production and endothelial cell migration in addition to increasing the expression of leukocyte adhesion molecules and thrombin receptors (Higashi, 2023). The observed increase in the serum oxLDL levels following CAFD feeding for 12 weeks can partly explain the noted reduction in NO levels, enhanced apoptotic pathways, and endothelial dysfunction. In the same context, the reduced levels of HDL-C level can be linked to endothelial dysfunction, which was observed in the rats fed CAFD.

FLE supplementation to the CAFD-fed rats induced a significant reduction in body weight and ameliorated dyslipidemia as witnessed by the significant reduction in serum TC, TG, LDL, and oxLDL concomitantly with significant elevation in serum HDL when compared to the CAFD-fed rats. Such results came in alignment with previous studies utilizing FLE (Sun et al., 2016; Draganescu et al., 2021; Sadat Masjedi et al., 2022) or SDG (Pilar et al., 2017; Guo et al., 2021)*.* The observed increase in serum HDL-C following FLE treatment can account for the reported improvement of the endothelial function manifested in the elevated NO and GSH levels, as well as the reduced TNF-α, IL-6, and MDA levels. This is supported by the previous findings that flaxseed can reduce inflammation and oxidative stress within the endothelium owing to the improved HDL-C function (Cockerill et al., 1995; Eren et al., 2014). Herein, the anti-dyslipidemic effect of FLE can be attributed to the fiber content in the flaxseed as the effective factor in reducing blood lipids (Torkan et al., 2015). Also, a previous work highlighted the ability of a high-fat diet containing SDG to significantly reduce the mRNA expression of sterol regulatory element binding protein-1c, which enhances the activity of cholesterol and fatty acid synthetase enzymes, which was probably responsible for the TC and TG-lowering effects by SDG (Fukumitsu et al., 2008). Another study pointed to the fact that flaxseed is one of the richest sources of the omega-3 fatty acid, alpha-linolenic acid, which has been demonstrated to lower serum lipid levels including TC, LDL, and TG in various animal models (Rodriguez-Leyva et al., 2010). Furthermore, it was found that flaxseed can interfere with fecal bile acid reabsorption, leading to its increased elimination and subsequent cholesterol utilization for bile acid synthesis. Thus, the increased excretion of bile acids can contribute to the lowering of serum cholesterol levels and the reduction of its accumulation in the endothelium, preserving the endothelial function (Kristensen et al., 2012; Higashi, 2023).

Interestingly, FLE supplementation did not show a significant difference in body weight or food consumption when compared to the control group, which supports the current controversy regarding the impact of FLE on food consumption and satiety as literature shows contradictory results in this issue (Zarei et al., 2022).

In the current study, rats fed with CAFD showed higher concentrations of serum TNF-α, CRP, and IL-6, and cardiac TNF-α and IFN-γ, versus a significant reduction in cardiac IL-10; which came in alignment with previous work (Zanchet et al., 2018). Fortunately, FLE supplementation to CAFD-fed rats showed a significant reduction of pro-inflammatory marker levels versus marked elevation of the anti-inflammatory cytokine, IL-10, which was consistent with previous studies (Palla et al., 2016; Morshedzadeh et al., 2021). This significant reduction of pro-inflammatory markers, following FLE supplementation, can be attributed to the ability of flaxseed lignan, SDG, to decrease pro-inflammatory markers due to the inhibition of transcription factors; JAK/STAT6 and NFκB (Wang et al., 2020; Yu et al., 2022).

The present work revealed that CAFD-fed rats exhibited a significant reduction in the nuclear Nrf2 protein expression levels versus a marked increase in the cytosolic Nrf2 content, caspase-3 activity, Bax levels, and MMP-9 mRNA expression. These findings refer to the CAFD-induced oxidative stress, inflammatory burden, and apoptotic response in the cardiac tissue of rats, which might contribute to the downregulation of Nrf2 via increasing Keap1 levels, enhancing the direction of Nrf2 for proteasomal degradation (Elrashidy, 2020). Singh et al. (2020) showed that TNF-α triggered the production of MMP-9 through the PKC signal transduction pathway. Thus, CAFD led to the acceleration of cell apoptosis within the heart tissue, which may be partly mediated through the intrinsic apoptosis pathway (Redza-Dutordoir and Averill-Bates, 2016).

Co-administration of FLE with CAFD showed a significant increase in the nuclear Nrf2 expression associated with a significant decline in the cytosolic Nrf2 protein expression, caspase-3 activity, Bax levels, and MMP-9 gene expression. This came in agreement with previous findings, in which SDG was effective in mitigating cardiomyocyte injury and death, and augmenting the antioxidant defense, thus, rationalizing the reported cardioprotective role of FLE (Puukila et al., 2015).

Another key pathogenic factor in various cardiovascular diseases is endothelial dysfunction. CAFD supplementation, in the present work, showed impaired endothelial function, as presented by elevated serum VCAM1 level. This came in agreement with a recent study, in which impaired endothelial function was observed following chronic high-fat diet consumption (Shabbir et al., 2022). The increase of VCAM1 may be attributed to hypercholesterolemia induced by the intake of a high-fat diet in this work. Moreover, VCAM1 elevated levels may be related to the increased TNF-α, as TNF-α levels were shown to be strongly correlated with atherogenesis, and its elevation results in direct impairment of endothelial function (Fan et al., 2019). Contrariwise, levels of VCAM1 were decreased upon FLE supplementation, which can be supported by a previous study showing that flaxseed oil reduced the expression of VCAM1 in the aortic tissue of apoE KO mice fed a high-fat diet to induce atherosclerosis (Han et al., 2015).

Hyperlipidemia and obesity are commonly associated with oxidative stress, which is capable of modifying vascular tone by impeding NO bioavailability and signaling (Čolak and Pap, 2021). During the oxidative stress state, excessive production of ROS reduces the bioactivity of NO due to its rapid oxidative inactivation by the ROS superoxide anion (Mendez-Albinana et al., 2022). In alignment, it was observed in the current work that CAFD supplementation induced a significant reduction of NO circulating levels.

The present study demonstrated significant upregulation of the cardiac mRNA expression and phospho-FOXO3a (Ser574) protein expression levels versus a significant reduction in the relative phospho-AMPK (Thr172) protein levels in CAFD-fed rats, which was consistent with previous observations (Cai et al., 2019; Fu et al., 2019). These findings might be explained by the report of Jeon (2016) that obesity, insulin resistance, and increased levels of the pro-inflammatory TNF-α can inhibit the phosphorylation of AMPK, and this can lead to exacerbation of oxidative stress, lipid accumulation, and increase in the levels of cytokines in the heart. Moreover, lipid-induced cardiac inflammation can suppress AMPK, suggesting the role of lipids as a nutrient stressor triggering inflammation. Wu et al. (2018) reported that hypoxia-induced apoptosis involved the increased expression of FOXO3a, which promoted the activation of apoptotic pathways. It was also reported that oxidative stress-induced apoptosis can be halted via the inhibition of FOXO3a (Wang et al., 2015). Interestingly, treatment of CAFD-fed rats with SDG, the phytochemical component of flaxseed, induced a significant increase in the phospho-AMPK (Thr172) and downregulated both the mRNA and protein expression of FOXO3a. Our findings were in agreement with those of previous studies illustrating the beneficial impact of flaxseed and SDG on AMPK and FOXO3a (Puukila et al., 2015; Sun et al., 2016). Therefore, SDG can modulate AMPK/FOXO3a pathway, and prevent oxidative stress-related pathogenesis, thus promoting its importance as a cardioprotective candidate.

To conclude, the co-administration of FLE with CAFD ameliorated the CAFD-induced oxidative stress, inflammation, and apoptosis, as well as the decreased AMPK expression, to which apelin/APJ contributed significantly. Accordingly, we conclude a protective role of FLE in CAFD-induced hyperlipidemic cardiovascular injury as FLE showed cardio- and vasculo-protective potentials that involve the regulation of the apelin/APJ signaling pathway. This may be attributed to FLE’s effectiveness in controlling cholesterolemic status and improving dyslipidemia and redox state. In addition to protection against cardiac injury and endothelial dysfunction, FLE modulated fibrosis by reducing the levels of PAI-1 and collagen percentage. Thus, FLE may be a novel therapeutic strategy for atherosclerosis prevention and cardiovascular injury.

## Figures and Tables

**Table:** 

**AC**	Atherogenic coefficient
**AI**	Atherogenic index
**AMPK**	AMP-activated protein kinase
**APJ**	Apelin receptor
**Bax**	Bcl-2-associated X protein
**CAFD**	Cafeteria diet
**CRP**	C-reactive protein
**CRR**	Cardiac risk ratio
**cTnI**	Cardiac troponin I
**ET-1**	Endothelin-1
**FLE**	Flaxseed lignan extract
**FOXO3a**	Forkhead box O3 a
**GSH**	Reduced glutathione
**H&E**	Hematoxylin and Eosin
**HDL-C**	High-density lipoprotein-cholesterol
**IFN-γ**	Interferon-gamma
**IL**	Interleukin
**LDL-C**	Low-density lipoprotein-cholesterol
**MDA**	Malondialdehyde
**MMP**	Matrix metalloproteinase
**NO**	Nitric oxide
**Nrf2**	Nuclear factor erythroid 2-related factor 2
**O.D.**	Optical density
**oxLDL**	Oxidized low-density lipoprotein
**PAI-1**	Plasminogen activator inhibitor-1
**PAS**	Periodic acid–Schiff
**PON1**	paraoxonase 1
**ROS**	Reactive oxygen species
**SDG**	Secoisolariciresinol diglucoside
**TC**	Total cholesterol
**TG**	Triglycerides
**TNF-α**	Tumor necrosis factor-alpha
**VCAM1**	Vascular cell adhesion molecule 1

## Data Availability

The original contributions presented in the study are included in the article/Supplementary Material, further inquiries can be directed to the corresponding author.
