# Supplementary material for: The cardioprotective effects of secoisolariciresinol diglucoside (flaxseed lignan) against cafeteria diet-induced cardiac fibrosis and vascular injury in rats: an insight into apelin/AMPK/FOXO3a signaling pathways
Source: Front Pharmacol. 2023 Jul 11;14:1199294. doi: 10.3389/fphar.2023.1199294 (PMC10367100; doi:10.3389/fphar.2023.1199294)
Supplement: Supplementary file 1 [file Image1.pdf]

Supplementary Material

The cardioprotective effects of secoisolariciresinol diglucoside (flaxseed lignan) against cafeteria diet-induced cardiac fibrosis and vascular injury in rats: an insight into apelin/AMPK/FOXO3a signaling pathways

Azza H. Abdelwahab[1], Amira M. Negm[1], Eman S. Mahmoud[2], Rania M. Salama[3]*, Mona F. Schaalan[4], Azza A.K. El-Sheikh[5], Basma K. Ramadan[1]

*** Correspondence:** Rania M. Salama: rania.salama@miuegypt.edu.eg

1 Supplementary Figures

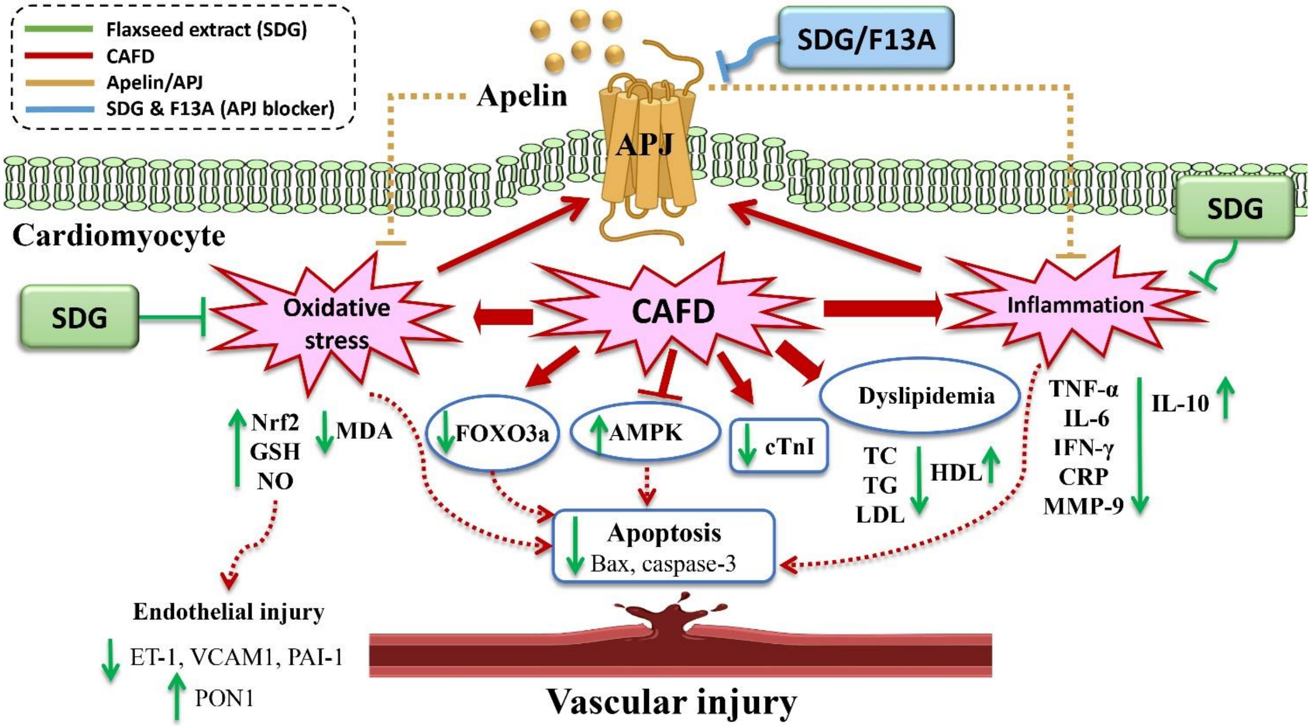

Supplementary Figure 1. Graphical abstract illustrating the predicted cardioprotective mechanisms of action of secoisolariciresinol diglucoside (SDG), the flaxseed lignan extract (FLE), against cafeteria diet (CAFD)-induced cardiac fibrosis and vascular injury in rats. F13A is an apelin-13 receptor blocker.
